# Unraveling the link between cardiorespiratory fitness and cancer: a state-of-the-art review

**DOI:** 10.1007/s11357-024-01222-z

**Published:** 2024-06-03

**Authors:** Setor K. Kunutsor, Leonard A. Kaminsky, Andrea Lehoczki, Jari A. Laukkanen

**Affiliations:** 1 Diabetes Research Centre, Leicester General Hospital, University of Leicester, Leicester, LE5 4WP UK; 2 Clinical Exercise Physiology, College of Health, Ball State University, Muncie, IN USA; 3 Department of Public Health, Semmelweis University, Budapest, Hungary; 4 Doctoral College, Health Sciences Program, Semmelweis University, Budapest, Hungary; 5 Department of Haematology and Stem Cell Transplantation, National Institute for Haematology and Infectious Diseases, South Pest Central Hospital, 1097 Budapest, Hungary; 6 Institute of Clinical Medicine, Department of Medicine, University of Eastern Finland, Kuopio, Finland; 7 Department of Medicine, Wellbeing Services County of Central Finland, Jyväskylä, Finland

**Keywords:** Cardiorespiratory fitness, Cancer, Mortality, Physical activity, Exercise, Mendelian randomization

## Abstract

Cardiorespiratory fitness (CRF) not only reflects an individual’s capacity to perform physical activities but also encapsulates broader effects on the basic biology of aging. This review aims to summarize the evidence on the influence of CRF on overall and site-specific cancer risks. It delves into the biological mechanisms through which CRF may exert its effects, explores the clinical implications of these findings, identifies gaps in the current evidence base, and suggests directions for future research. The synthesis of findings reveals that higher CRF levels (general threshold of > 7 METs) are consistently associated with a reduced risk of a range of cancers, including head and neck, lung, breast, gastrointestinal, particularly pancreatic and colorectal, bladder, overall cancer incidence and mortality, and potentially stomach and liver, bile duct, and gall bladder cancers. These inverse associations between CRF and cancer risk do not generally differ across age groups, sex, race, or adiposity, suggesting a universal protective effect of CRF. Nonetheless, evidence linking CRF with skin, mouth and pharynx, kidney, and endometrial cancers is limited and inconclusive. Conversely, higher CRF levels may be potentially linked to an increased risk of prostate cancer and hematological malignancies, such as leukemia and myeloma, although the evidence is still not conclusive. CRF appears to play a significant role in reducing the risk of several cancers through various biological mechanisms, including inflammation reduction, immune system enhancement, hormonal regulation, and metabolic improvements. Overall, enhancing CRF through regular physical activity offers a vital, accessible strategy for reducing cancer risk and extending the health span. Future research should aim to fill the existing evidence gaps regarding specific cancers and elucidate the detailed dose–response relationships between CRF levels and cancer risk. Studies are also needed to elucidate the causal relationships and mechanistic pathways linking CRF to cancer outcomes.

**Supplementary Information:**

The online version contains supplementary material available at 10.1007/s11357-024-01222-z.

## Introduction

Cancer remains a leading cause of morbidity and mortality worldwide, presenting a significant public health burden with millions of new cases and deaths annually [1]. The epidemiology of cancer is complex, influenced by a myriad of risk factors ranging from genetic predispositions to lifestyle choices [2]. Among these, physical activity (PA) emerges as a modifiable risk factor, with a growing body of evidence underscoring its protective role against various types of cancer. Regular PA is associated with a lower risk of colon, breast, and endometrial cancers, among others [3, 4], highlighting its importance in cancer prevention strategies.

Cardiorespiratory fitness (CRF), a measure of the body's ability to supply oxygen to the muscles during sustained PA [5, 6], serves as a direct outcome of regular PA. CRF not only reflects physical health but is also a strong risk indicator and predictor of several adverse cardiovascular outcomes [5, 7–15]. The nature, magnitude, and specificity of the relationships between CRF and adverse cardiovascular outcomes have been described as inverse, graded, and independent of established risk factors [5, 7–10, 16] and manifest similarly across different demographic subgroups, including varying age, sex, and race spectra [17, 18]. Evidence suggests that the protective effect of higher CRF levels is so strong that it can substantially modify, mitigate, or negate the adverse effects of other risk factors [19–24]. High CRF levels have also been shown to potentiate the beneficial effects of protective factors such as frequent sauna baths [25–29]. Given the substantial evidence demonstrating the importance of CRF as an important clinical tool, the American Heart Association in 2016 published a Scientific Statement suggesting that CRF be considered a clinical vital sign that should be assessed together with other established risk factors [5, 6]. Despite its significance, CRF has yet to be incorporated into standard cardiovascular risk prediction models, underlining a gap between its recognized importance and clinical application. Its role extends beyond cardiovascular disease prevention, encompassing a potential protective effect against the development of several non-vascular outcomes [30–32] including cancers [33].

There is a substantial body of evidence linking higher CRF levels with reduced risk of overall and site-specific cancers [33, 34]. However, there have been discrepancies in the literature. For instance, higher CRF levels have been linked to an increased risk of prostate and malignant skin cancers in some reports [33–35], whereas others have found no association between CRF and some cancer types [33, 35, 36]. This inconsistency necessitates a comprehensive summary of the evidence to better understand CRF’s overall impact on overall cancer and site-specific cancers. Given the significant public health implications of cancer, this review aims to synthesize the extensive observational evidence on the influence of CRF on overall and site-specific cancer risks. It will delve into the biological mechanisms through which CRF may exert its effects, explore the health, clinical, and policy implications of these findings, identify gaps in the current evidence base, and suggest directions for future research. It also reviews evidence on the genetic relationships between CRF and cancers. Addressing these aspects is essential for advancing our understanding of CRF’s (via regular PA and/or exercise) role in cancer prevention and management, thereby contributing to broader public health strategies and guidelines aimed at mitigating the cancer burden.

## Methods

A search of MEDLINE and Embase was conducted up to March 2024 for observational (including prospective cohort, nested case–control, case-cohort, or retrospective cohort studies) and interventional studies with a particular focus on systematic reviews and meta-analyses of these study designs if they were available, using the hierarchy of evidence [37]. Search terms or keywords related to cardiorespiratory fitness (e.g., “aerobic fitness,” “cardiovascular fitness,” “aerobic capacity,” “cardio fitness,” “VO2max,” and “VO2peak”) and cancer (e.g., “cancer,” “lung cancer,” “colorectal cancer,” “digestive cancer,” skin cancer,” “prostate cancer,” “cancer mortality,” and “cancer recurrence”) were combined. The review was restricted to studies conducted in the human population, reported in English and adults. Cross-sectional studies were not included because they do not address the issue of temporality. To assess the genetic (causal) associations between CRF and cancer outcomes, we conducted a separate search of Mendelian randomization (MR) studies on CRF and cancer.

## Terminologies for CRF and other related measures

To avoid any confusion, there is a need to define and clarify some related terminologies which will feature a lot in this review – “CRF,” “physical activity,” and “exercise.” Although “physical activity” and “exercise” are terms that are commonly used interchangeably, they are not necessarily the same. PA is defined as any skeletal muscle movement that increases energy expenditure beyond the resting level and includes exercise, leisure time activity, and usual occupational and/or domestic activity [38]. In contrast, exercise is a subcategory of PA and represents intentional PA that is designed to improve or maintain physical fitness and can include aerobic, high-intensity interval, or resistance training [39]. CRF is a measurable health outcome of PA and exercise training. It is defined as the capacity of the cardiovascular and respiratory systems to supply oxygen to the skeletal muscles during PA and/or exercise training [5, 40]. CRF is also referred to as aerobic capacity, maximal oxygen uptake (VO2max), or peak oxygen uptake (VO2peak), depending on the objective method of measurement. A wide range of methods are used to assess CRF, and these range from directly measured during cardiopulmonary exercise testing (CPX) using a treadmill or cycle ergometer to estimation from exercise tests or attained workload and non-exercise prediction equations [5, 41, 42]. VO2max or VO2peak assessed during CPX is considered the gold standard for assessing CRF [5]. VO2max is the maximum amount of oxygen that an individual can utilize during intense or maximal exercise. VO2max is reached when VO2 remains steady despite an increase in workload, indicating the individual’s maximum capacity for oxygen use during aerobic exercise [43, 44]. VO2peak, on the other hand, refers to the highest value of VO2 achieved during a graded exercise test when a true VO2max cannot be determined because the test subject fails to meet the criteria for VO2max (such as a plateau in oxygen uptake with increasing workload) [45]. It is essential to note that the majority of studies employ indirect methods or non-exercise algorithms for estimating CRF rather than the gold standard measure. These non-exercise-based algorithms can conveniently estimate CRF in a rapid, inexpensive, and reasonably accurate way when used for large population settings [5, 6]. However, estimating CRF rather than the use of the gold standard measure is associated with limitations, which can include (i) underestimation and overestimation of CRF at the top and bottom ends of the distribution, respectively, and (ii) variability in assessment methodologies of the input variables (e.g., heart rate) used to estimate CRF; hence, not all equations are suitable for particular populations [5, 6]. As a result, comparing and interpreting CRF values can be challenging. CRF is commonly expressed as mL/kg/min or metabolic equivalents (METs). The unit of METs is a measure of absolute intensity and reflects energy expenditure during rest (which approximates 3.5 mL/kg/min for the average adult) [46]. CRF generally declines with age; it peaks between the 2nd and 4th decade and then inevitably declines in both sedentary and trained individuals as well [47]. The average rates of decline per decade over a 6-decade period have been reported to range from 13.5%, 4.0 mL/kg/min to 16.4%, 4.3 mL/kg/min [48]. Although no global standards have been developed for CRF, there are indications that values may vary across countries [48].

CRF, while primarily a measure of the capacity of the cardiovascular and respiratory systems to supply oxygen to muscles during PA, also serves as a valuable proxy for the broader biological impacts of exercise. These include a range of exercise-induced physiological responses such as increased shear stress-mediated endothelial and vascular effects [49–52], angiogenesis [53–55], mitochondrial enhancements [56–58], and the release of exerkines [59–61], which collectively contribute to the systemic health benefits of regular PA. Moreover, the anti-inflammatory [56–58, 62] and neuroendocrine [63–65] effects of PA and exercise further substantiate the link between high CRF levels and its impact on cancer morbidity and mortality. The relationship between exercise, improved lung and respiratory function, and other systemic effects suggests that higher CRF levels might correlate with greater overall exercise benefits. Hence, CRF provides a relatively straightforward, meaningful measure of the impact of exercise, albeit not directly indicative of causality. This makes CRF an essential, although not exhaustive, metric for understanding how PA and exercise could influence cancer outcomes, reflecting the complex interplay between physical fitness and disease modulation.

## CRF and cancer outcomes in the general population

### Skin cancer

The relationship between CRF and skin cancer appears to be complex. In a cohort of 1997 healthy Norwegian men aged 40–59 years at inclusion, Robsahm and colleagues [66] in 2017 demonstrated no significant evidence of an association between CRF and skin cancer: (HR = 2.19, 95% CI, 0.99–4.96) for melanoma and (HR = 1.20, 95% CI, 0.55–2.60) for non-melanoma comparing the top vs. bottom tertiles of CRF [66]. In a 2017 prospective evaluation of the Veterans Exercise Testing Study (VETS) cohort, Vainshelboim and colleagues [67] showed no strong evidence of an association between CRF and skin cancer incidence. However, Onerup and colleagues [33] in their 2023 study of Swedish military conscripts showed that higher CRF was linearly associated with a higher hazard of being diagnosed with malignant skin cancer (HR = 1.13, 95% CI, 1.09–1.17) and (HR = 1.31, 95% CI, 1.27–1.36) for moderate (standardized score 6–7) and high CRF (standardized score 8–9) categories, respectively, compared to the lowest CRF (standardized score 1–5) category. In a related study by the same group [36], there was a linear protective association between CRF and 5-year mortality after malignant skin cancer diagnosis: (HR = 0.86, 95% CI, 0.73–1.01) and (HR = 0.80, 95% CI, 0.67–0.95) for moderate and high CRF categories, respectively, compared to the lowest CRF category.

Limited prospective evidence suggests a complex relationship between CRF and skin cancer; the evidence is not conclusive.

### Central nervous system cancer

Robsahm and colleagues [66] in their 2017 study of healthy Norwegian men showed no significant evidence of an association between CRF and central nervous system (CNS) cancer. Onerup and colleagues [33] in their 2023 study of Swedish military conscripts showed no association between CRF and the risk of CNS cancer. In a related study by the same group [36], there was no evidence of an association between CRF and 5-year mortality after CNS cancer diagnosis.

There appears to be a consistent lack of association between CRF and the risk of CNS cancer, but this evidence is based on a limited number of studies.

### Head and neck cancer

Onerup and colleagues [33] showed that higher CRF was linearly associated with a lower risk of developing cancer in the head and neck: (HR = 0.87, 95% CI, 0.79–0.95) and (HR = 0.81, 95% CI, 0.74–0.90) for moderate and high CRF categories, respectively, compared to the lowest CRF category. In another study by the same group [36], there was a linear inverse association between CRF and 5-year mortality after head and neck cancer diagnosis: (HR = 0.74, 95% CI, 0.61–0.91) and (HR = 0.68, 95% CI, 0.54–0.85) for moderate and high CRF categories, respectively, compared to the lowest CRF category.

The evidence on the association between CRF and head and neck cancer is limited but suggests a protective association.

#### Thyroid cancer

Onerup and colleagues [33] in their 2023 study of Swedish military conscripts showed no association between CRF and the risk of thyroid cancer (HR = 1.01, 95% CI, 0.83–1.24) comparing high vs. low CRF categories. Similarly, in a related study by the same group [36], there was no evidence of an association between CRF and 5-year mortality after thyroid cancer diagnosis.

There appears to be no significant association between CRF and the risk of thyroid cancer, but this evidence is based on a limited number of studies.

### Lung cancer

Lakoski and colleagues [68] in 2015 conducted a prospective evaluation of the Cooper Center Longitudinal Study (CCLS) and showed higher midlife CRF to be associated with a decreased risk of lung cancer (HR = 0.57, 95% CI, 0.41–0.81) and (HR = 0.45, 95% CI, 0.29–0.68) for moderate and high CRF categories, respectively, compared to the lowest CRF category. In a 2016 evaluation of the Finnish Kuopio Ischemic Heart Disease (KIHD) cohort comprising 2305 men with no history of cancer at baseline, Pletnikoff and colleagues [69] showed that decreased CRF levels were associated with an increased risk of lung cancer (HR = 2.88, 95% CI, 1.14–7.22) comparing the bottom (≤ 7 METs) vs. top (> 10 METs) quartiles of CRF. Robsahm and colleagues [66] in their 2017 study of healthy Norwegian men showed evidence of an inverse association between CRF and lung cancer (HR = 0.39, 95% CI, 0.23–0.66) comparing high vs. low CRF categories. Pozuelo-Carrascosa and colleagues [34] in a 2019 meta-analysis of 10 prospective studies showed that CRF was inversely associated with the risk of lung cancer (HR = 0.53, 95% CI, 0.39–0.68) and (HR = 0.52, 95% CI, 0.42–0.61) for intermediate and highest CRF categories, respectively, compared to the lowest CRF category. In a 2019 prospective evaluation of the VETS cohort, Vainshelboim and colleagues [70] showed that higher CRF was associated with a lower risk of lung cancer incidence in former smokers and lung cancer mortality in current smokers. For lung cancer incidence in former smokers: (HR = 0.49, 95% CI, 0.25–0.97) and (HR = 0.23, 95% CI, 0.08–0.66) for moderate (5–10 METs) and high CRF (> 10 METs) categories, respectively, compared to the lowest CRF (< 5 METs) category. For lung cancer mortality in current smokers: (HR = 0.16, 95% CI, 0.06–0.40) and (HR = 0.15, 95% CI, 0.05–0.50) for moderate and high CRF categories, respectively, compared to the lowest category. In a 2023 evaluation of the National Institutes of Health-American Association of Retired Persons (NIH-AARP) diet and health cohort study that included 402,548 participants free from cancer at baseline, Vainshelboim and colleagues [71] showed that CRF was not associated with the risk of lung cancer in both men and women. Onerup and colleagues [33] in their 2023 study of Swedish military conscripts showed that higher CRF was linearly associated with a lower risk of lung cancer (HR = 0.58, 95% CI, 0.51–0.66) comparing high vs. low CRF categories. In a similar study by Onerup and colleagues [36], there was a linear inverse association between CRF and 5-year mortality after lung cancer diagnosis: (HR = 0.83, 95% CI, 0.73–0.94) and (HR = 0.79, 95% CI, 0.68–0.91) for moderate and high CRF categories, respectively, compared to the lowest CRF category. Ekblom-Bak and colleagues [72] in a 2023 prospective cohort analysis of ~ 180,000 Swedish men showed that higher CRF levels were associated with a lower risk of lung cancer mortality (HR = 0.41, 95% CI, 0.17–0.99) comparing the highest (> 13 METs) vs. lowest CRF (≤ 7 METs) categories; there was no strong evidence of an association for lung cancer incidence [72]. Watts and colleagues [73] in their recent 2024 evaluation of the UK Biobank comprising 72,572 participants showed no evidence of an association between CRF and lung cancer risk. All studies reviewed accounted for smoking status in their analyses.

In summary, a significant body of robust research suggests that CRF is inversely associated with the risk of lung cancer incidence and mortality, independently of smoking status.

### Breast cancer

In the first prospective evaluation of CRF and breast cancer risk in 2009, Peel and colleagues [74] used the Aerobics Center Longitudinal Study (ACLS) comprising 14,811 women with no prior breast cancer history and showed that CRF was associated with a reduced risk of breast cancer mortality in a dose–response fashion: (HR = 0.67, 95% CI, 0.35–1.26) and (HR = 0.45, 95% CI, 0.22–0.95) for intermediate and highest CRF categories, respectively, compared to the lowest CRF category. The association was not modified by age, body mass index (BMI), and use of oral contraceptives or estrogen. The dose–response analysis showed that women with a CRF < 8 METs had a nearly three-fold higher risk of dying of breast cancer compared with those with higher CRF levels (≥ 8 METs) [74]. In 17,840 cancer-free postmenopausal women with a CRF assessment from the UK Biobank, Christensen and colleagues [75] in 2023 showed that high CRF was associated with a 24% lower risk of breast cancer (HR = 0.76, 95% CI, 0.60–0.97); this protective association was driven by women with elevated fat [75]. In a 2023 evaluation of the NIH-AARP diet and health cohort study that included 402,548 participants free from cancer at baseline, Vainshelboim and colleagues [71] showed that higher CRF was associated with a reduced risk of breast cancer (HR = 0.89, 95% CI, 0.82–0.96) and (HR = 0.88, 95% CI, 0.79–0.99) for moderate (6.1–8.2 METs) and high CRF (> 8.2 METs) categories, respectively, compared to the lowest CRF (< 6.1 METs) category. In 46,968 cancer-free adults who participated in the Norweigan Trøndelag Health Study (HUNT study), Wang and colleagues [76] in 2023 showed no evidence of an association between CRF and breast cancer incidence in women. Katsaroli and colleagues [77] in a 2024 evaluation of the ETHOS cohort comprising of 44,463 women showed that CRF was associated with breast cancer risk in an inverse and graded manner: (HR = 0.93, 95% CI, 0.90–0.95) per one-MET increase in CRF and (HR = 0.82, 95% CI, 0.70–0.96), (HR = 0.69, 95% CI, 0.58–0.82), and (HR = 0.60, 95% CI, 0.47–0.75) for low-fit, moderate-fit, and fit women, respectively, compared to the least-fit category. The associations were similar across race categories [77]. Watts and colleagues [73] recently showed that a one-MET increase in CRF was associated with a 4% reduction in breast cancer (HR = 0.96, 95% CI, 0.92–0.99), but the association was significantly attenuated on adjustment for BMI.

The link between CRF and breast cancer suggests a protective association, with several studies indicating that higher levels of CRF may reduce the risk of developing breast cancer.

### Gastrointestinal cancer

In a 2023 prospective evaluation of the VETS cohort, Vainshelboim and Myers [78] showed that higher CRF was associated with a lower risk of digestive system cancer incidence in the entire cohort of men (HR = 0.94, 95% CI, 0.91–0.98 per 1-MET increase), particularly in those < 60 years (HR = 0.91, 95% CI, 0.85–0.97 per 1-MET increase), never smokers (HR = 0.91, 95% CI, 0.83–1.00 per 1-MET increase), and current smokers (HR = 0.91, 95% CI, 0.84–0.99 per 1-MET increase). There was no association in men ≥ 60 years old and among former smokers.

Findings based on a single study suggest a protective association between CRF and digestive system cancer incidence.

#### Mouth and pharynx cancer

In a cohort of 1997 healthy Norwegian men, Robsahm and colleagues [66] in 2017 demonstrated no significant evidence of an association between CRF and cancer of the mouth or pharynx.

Findings based on a single study suggest no evidence of an association between CRF and mouth and pharynx cancer.

#### Esophageal cancer

Robsahm and colleagues [66] showed no significant evidence of an association between CRF and cancer of the esophagus in apparently healthy Norwegian men. Onerup and colleagues [33] in their 2023 study of Swedish military conscripts showed that higher CRF was linearly associated with a lower risk of cancer of the esophagus (HR = 0.61, 95% CI, 0.50–0.74) comparing high vs. low CRF categories. Similarly, in a related study by the same group [36], there was no evidence of an association between CRF and 5-year mortality after esophageal cancer diagnosis.

The evidence on the relationship between CRF and esophageal cancer is limited and not conclusive.

#### Stomach cancer

Robsahm and colleagues [66] in their 2017 study of healthy Norwegian men showed no significant evidence of an association between CRF and cancer of the stomach. Onerup and colleagues [33] in their 2023 study of Swedish military conscripts showed that higher CRF was linearly associated with a lower risk of stomach cancer (HR = 0.79, 95% CI, 0.67–0.94) comparing high vs. low CRF categories. In a study by the same group [36], there was an inverse association between CRF and 5-year mortality after stomach cancer diagnosis (HR = 0.78, 95% CI, 0.62–0.99) comparing high vs. low CRF categories.

Limited evidence suggests that CRF might be protective of stomach cancer.

#### Pancreatic cancer

Robsahm and colleagues [66] in their study showed evidence of an inverse association between CRF and pancreatic cancer (HR = 0.32, 95% CI, 0.10–1.00) comparing high vs. low CRF categories. Onerup and colleagues [33] in their 2023 study of Swedish military conscripts showed modest evidence that higher CRF levels might be linearly associated with a lower risk of pancreatic cancer (HR = 0.88, 95% CI, 0.76–1.01) comparing high vs. low CRF categories. Similarly, in a related study by the same group [36], there was some evidence of an inverse association between CRF and 5-year mortality after pancreatic cancer diagnosis (HR = 0.85, 95% CI, 0.72–1.01) comparing high vs. low CRF categories.

There is consistent evidence of a protective association between CRF and the risk of pancreatic cancer, but this is based on a limited number of studies.

#### Liver, bile ducts, and gall bladder cancer

Robsahm and colleagues [66] showed no significant evidence of an association between CRF and liver, bile ducts, and gall bladder cancer in men. Onerup and colleagues [33] in their 2023 study of Swedish military conscripts showed that higher CRF was linearly associated with a lower risk of liver, bile ducts, and gall bladder cancer (HR = 0.60, 95% CI, 0.51–0.71) comparing high vs. low CRF categories. In a related study by Onerup and colleagues [36], there was evidence of an inverse association between CRF and 5-year mortality after liver, bile ducts, and gall bladder cancer diagnosis: (HR = 0.83, 95% CI, 0.72–0.97) and (HR = 0.84, 95% CI, 0.71–1.01) for moderate and high CRF categories, respectively, compared to the lowest CRF category.

Limited evidence suggests that CRF might be protective of liver, bile ducts, and gall bladder cancer.

#### Colorectal cancer

Robsahm and colleagues [66] in their 2017 study of healthy Norwegian men showed no significant evidence of an association between CRF and cancer of the colon or rectum. In a 2019 dose–response evaluation of 73,259 UK Biobank participants, Steell and colleagues [79] showed that each one-MET higher CRF was associated with a lower risk for colorectal cancer (HR = 0.96, 95% CI, 0.92–1.00); furthermore, the risk for colorectal cancer decreased linearly beyond 8 METs. Pozuelo-Carrascosa and colleagues [34] in a 2019 meta-analysis of 10 prospective studies showed that CRF was inversely associated with the risk of colorectal cancer (HR = 0.74, 95% CI, 0.55–0.93) and (HR = 0.77, 95% CI, 0.62–0.92) for intermediate and highest CRF categories, respectively, compared to the lowest CRF category. In a 2023 evaluation of the NIH-AARP diet and health cohort study that included 402,548 participants free from cancer at baseline, Vainshelboim and colleagues [71] showed higher CRF was independently associated with a lower risk of colorectal cancer risk in men but not in women: (HR = 0.70, 95% CI, 0.59–0.84) and (HR = 0.89, 95% CI, 0.71–1.10), respectively, comparing higher vs. lower categories of CRF. Onerup and colleagues [33] in their 2023 study of Swedish military conscripts showed that higher CRF was linearly associated with a lower risk of colon cancer, with no evidence of an association for rectal cancer: (HR = 0.82, 95% CI, 0.75–0.90) and (HR = 0.95, 95% CI, 0.85–1.05), respectively, comparing high vs. low CRF categories. In a related study by the same group [36], there was no evidence of an association between CRF and 5-year mortality after colon cancer diagnosis, but there was an inverse association between CRF and 5-year mortality rectal cancer (HR = 0.79, 95% CI, 0.64–0.97) comparing high vs. low CRF categories. Ekblom-Bak and colleagues [72] in a 2023 prospective cohort analysis of ~ 180,000 Swedish men showed that higher CRF levels were associated with a lower risk of colon cancer incidence in a linear dose–response manner: (HR = 0.72, 95% CI, 0.53–0.96) and (HR = 0.63, 95% CI, 0.41–0.98) for moderate (> 10–13 METs) and high CRF (> 13 METs) categories, respectively, compared to the lowest CRF (≤ 7 METs) category; colon cancer incidence decreased continuously across the CRF range 7–16 METs [72]. There was no strong evidence of an association for colon cancer mortality [72]. Watts and colleagues [73] in their 2024 evaluation of the UK Biobank showed that a one-MET increase in CRF was associated with a 6% reduction in colorectal cancer (HR = 0.94, 95% CI, 0.90–0.99), but the association was significantly attenuated on adjustment for BMI.

A consistent body of evidence suggests a protective association between CRF and the risk of colorectal cancer.

### Genitourinary cancer

#### Prostate cancer

The relationship between CRF and prostate cancer is controversial (Supplementary information). In the first prospective evaluation of CRF and prostate cancer risk, Oliveria and colleagues [80] in 1996 showed that higher CRF levels were associated with a reduced risk of prostate cancer (HR = 0.26, 95% CI, 1.10–0.63) comparing the top vs. bottom quartiles of CRF. In a 2011 evaluation of the ACLS cohort, Byun and colleagues [81] showed evidence of an increased risk of prostate cancer associated with high CRF levels: (HR = 1.68, 95% CI, 1.13–2.48) and (HR = 1.74, 95% CI, 1.15–2.62) for moderate and high CRF categories, respectively, compared to the lowest CRF category. Lakoski and colleagues [68] in 2015 conducted a prospective evaluation of the CCLS and showed higher midlife CRF to be associated with an increased risk of prostate cancer (HR = 1.22, 95% CI, 1.02–1.46) comparing high vs. low CRF categories. Robsahm and colleagues [66] in their 2017 study of healthy Norwegian men showed no significant evidence of an association between CRF and prostate cancer. In a 2019 dose–response evaluation of 73,259 UK Biobank participants, Steell and colleagues [79] showed that high CRF (> 10 METs) was associated with a greater incidence of prostate cancer (HR = 1.16, 95% CI, 1.02–1.32) compared with average CRF. In a 2020 evaluation of a prospective cohort comprising 699,125 Swedish military conscripts, Crump and colleagues [82] showed that high CRF in late adolescence was associated with increased future risk of prostate cancer, but neither with risk of aggressive prostate cancer nor prostate cancer mortality: (HR = 1.10, 95% CI, 1.03–1.19) for any prostate cancer comparing high vs. low CRF levels. In a 2021 retrospective cohort analysis of the Henry Ford Exercise Testing (FIT) Project, Reiter-Brennan and colleagues [83] evaluated whether CRF was associated with prostate cancer screening, incidence, or mortality. Their results showed that compared with men who had low CRF (< 6 METs), those with high CRF (≥ 12 METs) had a higher risk of prostate-specific antigen (PSA) screening (incident rate ratio = 1.29, 95% CI, 1.25–1.33), higher prostate cancer incidence in men aged > 55 years (HR = 1.80, 95% CI, 1.27–2.54), and 60% lower risk of prostate cancer mortality (HR = 0.40, 95% CI, 0.19–0.86) [83]. Kunutsor and colleagues [35] in 2021 assessed the association of CRF with prostate cancer risk using the Finnish KIHD cohort study and a systematic review of 8 population-based prospective studies. Their primary data analysis and review of previous studies showed no evidence of an association between CRF and prostate cancer risk [35]. However, the authors noted that studies which reported positive associations had short follow-up durations (< 10 years); it was concluded that these findings could be attributed to increased screening and detection as well as reverse causation bias [35]. Ekblom-Bak and colleagues [72] in a 2023 prospective cohort analysis of ~ 180,000 Swedish men showed that moderate CRF (10–13 METs) but not high CRF (> 13 METs) levels were associated with a higher risk of prostate cancer incidence (HR = 1.18, 95% CI, 1.02–1.38) compared with very low CRF (≤ 10 METs) levels; high CRF levels were associated with a lower risk of prostate cancer mortality in a dose–response manner – The risk decreased continuously across the range 7–13 METs. In a 2023 evaluation of the NIH-AARP diet and health cohort study that included 402,548 participants free from cancer at baseline, Vainshelboim and colleagues [71] showed weak evidence higher CRF might be associated with increased prostate cancer incidence (HR = 1.09, 95% CI, 1.00–1.20) comparing higher (> 10.9 METs) vs. lower categories (< 8.9 METs) of CRF. In 46,968 cancer-free adults who participated in the HUNT study, Wang and colleagues [76] in 2023 reported modest evidence of an inverse association between CRF and prostate cancer: (HR = 0.85, 95% CI, 0.72–1.02) comparing the highest vs. lowest CRF categories. Onerup and colleagues [33] in their 2023 study of Swedish military conscripts showed that higher CRF was associated with an increased risk of prostate cancer (HR = 1.07, 95% CI, 1.03–1.12) comparing high vs. low CRF categories. In a related study by the same group [36], there was no evidence of an association between CRF and 5-year mortality after prostate cancer. A 2024 study by Bolam and colleagues [84] involving over 57,000 employed Swedish men found that changes in CRF were inversely associated with the risk of prostate cancer incidence but not prostate cancer mortality. Specifically, an increase in annual CRF by > 3% was linked to a 35% lower risk of developing prostate cancer (HR = 0.65, 95% CI, 0.49–0.86).

The evidence on the relationship between CRF and prostate cancer is not consistent, but the majority of studies report higher CRF levels to be linked to an increased risk of prostate cancer.

#### Kidney cancer

Robsahm and colleagues [66] in their study showed no significant evidence of an association between CRF and kidney cancer in apparently healthy Norwegian me. Onerup and colleagues [33] in their 2023 study of Swedish military conscripts showed that higher CRF was linearly associated with a decreased risk of developing kidney cancer (HR = 0.80, 95% CI, 0.70–0.90) comparing high vs. low CRF categories. In a related study by the same group [36], there was no evidence of an association between CRF and 5-year mortality after kidney cancer diagnosis.

The evidence on the relationship between CRF and kidney cancer is limited and not conclusive.

#### Bladder cancer

Robsahm and colleagues [66] in their study showed evidence of an inverse association between CRF and bladder, ureter, and urethra cancer (HR = 0.40, 95% CI, 0.21–0.74) comparing high vs. low CRF categories. Onerup and colleagues [33] in their 2023 study of Swedish military conscripts showed that higher CRF was linearly associated with a decreased risk of developing bladder cancer (HR = 0.90, 95% CI, 0.81–1.00) comparing high vs. low CRF categories. In a related study by Onerup and colleagues [36], there was evidence of an inverse association between CRF and 5-year mortality after bladder cancer diagnosis: (HR = 0.90, 95% CI, 0.67–1.19) and (HR = 0.71, 95% CI, 0.51–0.98) for moderate and high CRF categories, respectively, compared to the lowest CRF category.

There is consistent evidence of a protective association between CRF and the risk of bladder cancer, but this is based on a limited number of studies.

#### Endometrial cancer

Watts and colleagues [73] in their 2024 evaluation of the UK Biobank showed that a one-MET increase in CRF was associated with a 19% reduction in endometrial cancer (HR = 0.81, 95% CI, 0.73–0.89); the association was attenuated on adjustment for BMI.

Findings based on a single study suggest no evidence of an association between CRF and endometrial cancer.

### Haematological malignancies

#### Leukemia

Robsahm and colleagues [66] in their 2017 study of healthy Norwegian men showed no significant evidence of an association between CRF and leukemia. Onerup and colleagues [33] in their 2023 study showed that higher CRF was associated with an increased risk of leukemia (HR = 1.14, 95% CI, 1.01–1.28) comparing high vs. low CRF categories. In a related study by the same group [36], there was no evidence of an association between CRF and 5-year mortality after leukemia diagnosis.

The evidence on the relationship between CRF and leukemia is limited and not conclusive.

#### Myeloma

Robsahm and colleagues [66] in their 2017 study of healthy Norwegian men showed no significant evidence of an association between CRF and myeloma. Onerup and colleagues [33] in their 2023 study showed that higher CRF was associated with an increased risk of myeloma (HR = 1.21, 95% CI, 1.03–1.44) comparing high vs. low CRF categories. In a related study by the same group [36], there was no evidence of an association between CRF and 5-year mortality after myeloma diagnosis.

The evidence on the relationship between CRF and myeloma is limited and not conclusive.

#### Hodgkin’s and non-Hodgkin’s lymphoma

There was no strong evidence of any associations of CRF with Hodgkin’s and non-Hodgkin’s lymphoma in the Swedish study by Onerup and colleagues [33]. Similarly, in a related study by the same group [36], there was no evidence of an association between CRF and 5-year mortality after Hodgkin’s and non-Hodgkin’s lymphoma diagnosis.

Findings based on a single study suggest no evidence of an association between CRF and Hodgkin’s and non-Hodgkin’s lymphoma.

### Overall cancer incidence and mortality

In a 2010 evaluation of the KIHD cohort comprising 2268 men with no history of cancer at baseline, Laukkanen and colleagues [85] showed that high CRF levels were associated with a decreased risk of overall cancer incidence and mortality (HR = 0.73, 95% CI, 0.56–0.97) for cancer incidence comparing the highest (> 9.5 METs) vs. lowest (< 8.3 METs) tertiles of CRF and (HR = 0.63, 95% CI, 0.40–0.97) for cancer mortality comparing the highest vs. lowest tertiles of CRF. Schmid and Leitzmann [86] in 2015 conducted a meta-analysis of 6 prospective cohort studies to evaluate the association between CRF and cancer mortality risk. Their results showed a strong, graded, inverse association of CRF with total cancer mortality: (RR = 0.80, 95% CI, 0.67–0.97) and (RR = 0.55, 95% CI, 0.47–0.65) for intermediate and highest CRF categories, respectively, compared to the lowest CRF category. The association was not attenuated on adjustment for adiposity. In a 2017 prospective evaluation of the VETS cohort, Vainshelboim and colleagues [67] showed that higher CRF was associated with a lower risk of total cancer incidence: (HR = 0.96, 95% CI, 0.95–0.98) per one-MET increase in CRF and (HR = 0.86, 95% CI, 0.74–0.99) and (HR = 0.74, 95% CI, 0.62–0.89) for moderate (5–10 METs) and high (> 10 METs) categories, respectively, compared to the lowest (< 5 METs) category. In a 2019 evaluation of the Ball state Adult fitness Longitudinal Lifestyle STudy cohort, Imboden and colleagues [87] showed an inverse relationship between the change in CRF over time and risk for cancer mortality; specifically, a 1 mL/kg/min increase in CRF was associated with a 14% risk reduction in cancer mortality (HR = 0.86, 95% CI, 0.77–0.96). In a 2019 dose–response evaluation of 73,259 UK Biobank participants, Steell and colleagues [79] showed evidence of a linear inverse association between CRF and cancer mortality for a CRF range of 6–14 METs. In a 2019 prospective evaluation of the VETS cohort, Vainshelboim and colleagues [70] showed that higher CRF was associated with lower risk of cancer mortality in current smokers who were diagnosed with lung cancer: (HR = 0.82, 95% CI, 0.71–0.95) per one-MET increase in CRF and (HR = 0.16, 95% CI, 0.06–0.40) and (HR = 0.15, 95% CI, 0.05–0.50) for moderate (5–10 METs) and high (> 10 METs) categories, respectively, compared to the lowest (< 5 METs) category. Pozuelo-Carrascosa and colleagues [34] in a 2019 meta-analysis of 10 prospective studies showed that CRF was inversely associated with the risk of overall cancer (HR = 0.86, 95% CI, 0.79–0.93) and (HR = 0.81, 95% CI, 0.75–0.87) for intermediate and highest CRF categories, respectively, compared to the lowest CRF category. In a 2021 meta-analysis of 13 prospective cohort studies, Ezzatvar and colleagues [88] showed a reduced risk of all-cause mortality among adults diagnosed with any cancer: (HR = 0.82, 95% CI, 0.69–0.99) per one-MET increase in CRF and (HR = 0.52, 95% CI, 0.35–0.77) comparing high vs. lower CRF categories. The association was not modified by baseline age [88]. In a 2022 dose–response meta-analysis of observational cohort studies, Han and colleagues [89] showed an inverse dose–response association between CRF and cancer mortality: (RR = 0.93, 95% CI, 0.91–0.96) per one-MET increase in CRF and (RR = 0.76, 95% CI, 0.69–0.84) and (RR = 0.57, 95% CI, 0.46–0.70) for intermediate and highest CRF categories, respectively, compared to the lowest CRF category. The association did not vary by sex, location, and CRF assessment methods [89]. In a 2023 evaluation of the NIH-AARP diet and health cohort study that included 402,548 participants free from cancer at baseline, Vainshelboim and colleagues [71] showed higher CRF was independently associated with lower risk of total cancer incidence in both sexes: (HR = 0.96, 95% CI, 0.94–0.97) and (HR = 0.95, 95% CI, 0.93–0.97) per one-MET increase in CRF for men and women, respectively. In 46,968 cancer-free adults who participated in the HUNT study, Wang and colleagues [76] in 2023 reported an inverse dose–response association between CRF and overall cancer incidence: (HR = 0.96, 95% CI, 0.90–1.01) and (HR = 0.85, 95% CI, 0.79–0.91) for intermediate and highest CRF categories, respectively, compared to the lowest CRF category. The association was not significantly modified by sex [76]. In a 2023 Swedish study of military conscripts by Onerup and colleagues [36], there was a linear inverse association between CRF and 5-year mortality after any cancer (HR = 0.85, 95% CI, 0.82–0.89) and (HR = 0.70, 95% CI, 0.67–0.74) for moderate and high CRF categories, respectively, compared to the lowest CRF category.

There is a consistent body of evidence showing that higher levels of CRF are associated with a reduced risk of developing overall cancer incidence and mortality.

## Cancer recurrence in individuals with a prior diagnosis of cancer

Although several studies have evaluated the associations between PA or exercise and cancer recurrence [90–93], our search of the literature did not identify any studies that focused solely on the relationship between CRF and cancer recurrence in individuals with a previous diagnosis of cancer.

## Evidence from Mendelian randomization studies

MR studies offer a powerful approach to assess the causal relationship between CRF and cancer risk. MR utilizes genetic variants as instrumental variables to estimate the effect of an exposure (in this case, CRF) on an outcome (cancer risk), aiming to overcome confounding factors and reverse causality issues inherent in observational studies. The evidence from MR studies on the causal relationship between CRF and cancer is still emerging. Our extensive review of the literature suggests that MR studies on the causal effects of CRF on cancer outcomes are limited. One of the challenges in directly linking CRF to cancer risk through MR studies is the identification of genetic variants that accurately represent CRF levels. We identified only one study that met the criteria. Watts and colleagues [73] employed the UK Biobank and independent genome-wide association study (GWAS) data from international consortia to explore the observational and genetic associations between CRF and several site-specific cancers. The genetic instrument employed for CRF included 14 fitness and 149 fitness and resting heart rate (RHR) genome-wide significant variants [73]. Given that RHR is inversely correlated with CRF in observational studies and decreases as a response to aerobic exercise training [94–96], RHR has been used as a proxy trait for fitness in genetic studies of CRF [97]. In the MR analyses, a 0.5 SD increase in genetically predicted VO2max fat-free mass was associated with a lower risk of breast cancer (OR = 0.92, 95% CI, 0.86–0.98). After adjusting for adiposity, which may both mediate and confound the relationship between CRF and cancer risk, the association was significantly attenuated. While direct evidence from MR studies on CRF and cancer risk is still developing, the approach holds promise for clarifying this complex relationship.

## Potential pathways underlying the association between CRF and cancer

The inconsistency in the findings for certain site-specific cancers may stem from several factors. These include differences in study populations, which can vary widely in age, sex, race, and genetic background. Additionally, study design elements such as sample size, follow-up duration, and the extent of adjustment for confounding variables also play relevant roles. Smaller sample sizes or shorter follow-up periods may not adequately capture the relationship between CRF and cancer outcomes. Moreover, studies that do not sufficiently adjust for confounders might report associations that could be attributed to these uncontrolled variables rather than to CRF itself.

**Fig. 1:**
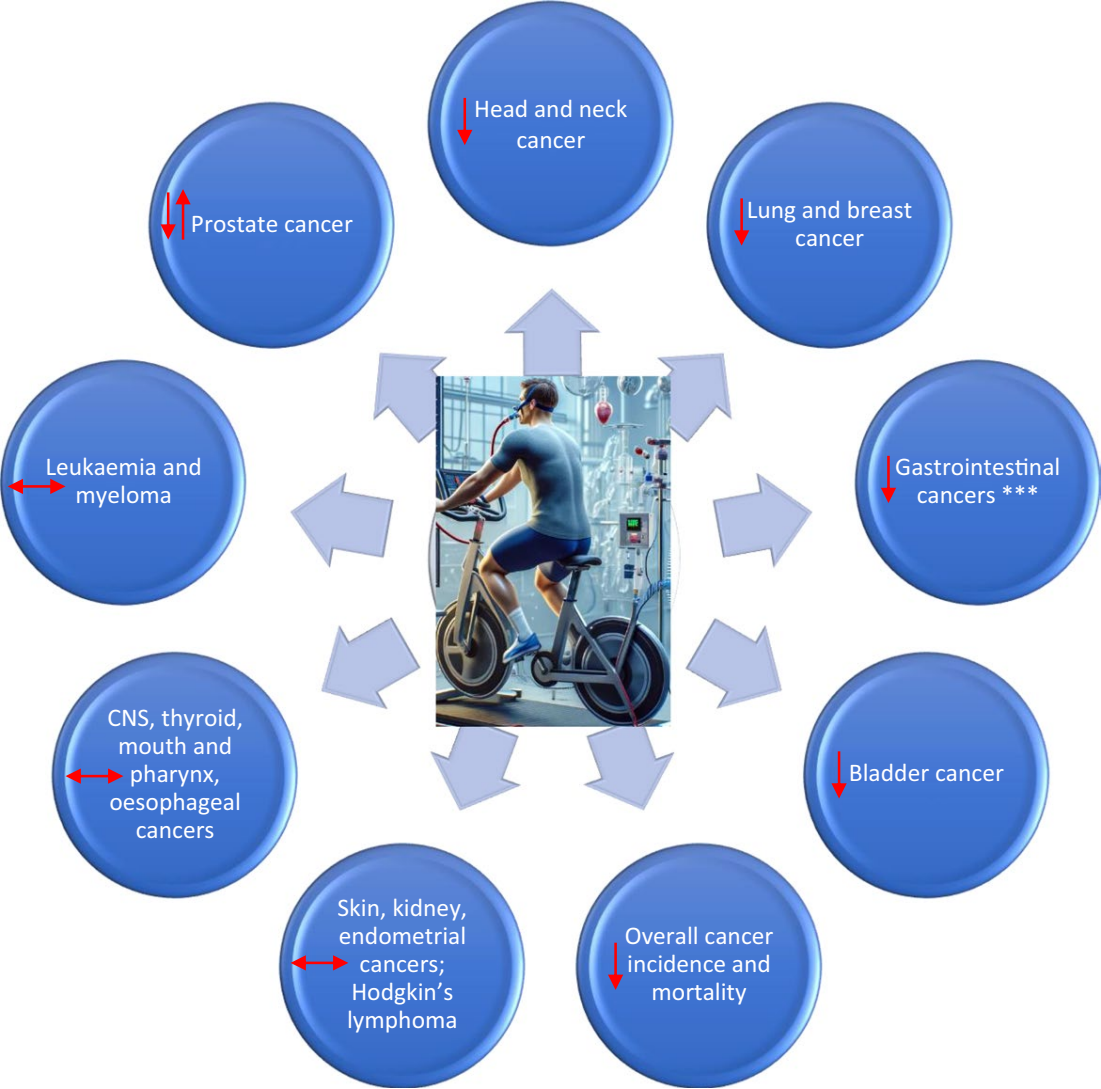
Cardiorespiratory fitness and cancer outcomes: summary of effects. CNS, central nervous system. ***For gastrointestinal cancers, these include particularly pancreatic and colorectal cancers and potentially stomach and liver, bile duct, and gall bladder cancers

**Fig. 2:**
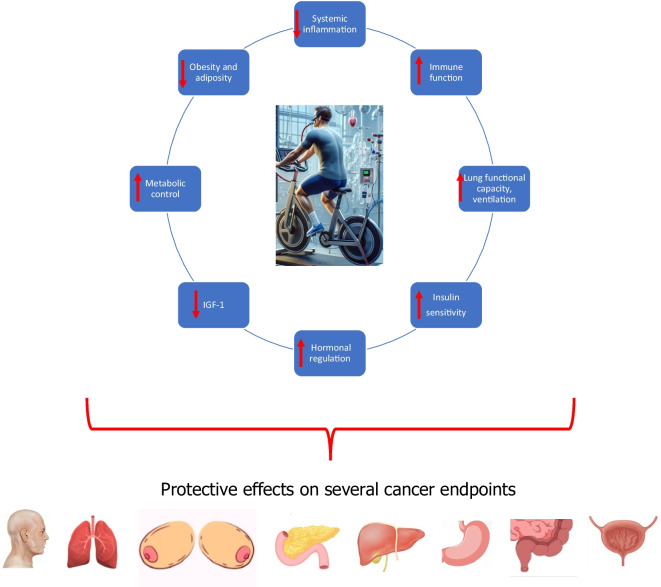
Proposed mechanistic pathways underlying the beneficial effects of CRF on cancer risk. IGF-1, insulin-like growth factor 1

The association between CRF and cancer risk involves complex biological mechanisms that may influence cancer development and progression across various types of cancer. CRF, often considered a proxy measure for the overall effects of PA and exercise, reflects not just physical endurance but also encapsulates the broader physiological changes brought about by PA and regular exercise. These changes include improved metabolic health, enhanced immune function, and reduced systemic inflammation, all of which can impact cancer etiology and progression. Importantly, various cancers have different etiologies and mechanistic pathways underlying their development. High levels of CRF are linked to a reduced risk of several cancers, including head and neck, lung, breast, gastrointestinal particularly pancreatic and colorectal, bladder, overall cancer incidence and mortality, and potentially stomach and liver, bile duct, and gall bladder cancers (Fig. 1). The protective effects of CRF on cancer can be attributed to multiple pathways (Fig. 2), further underscoring the role of regular PA and/or exercise, which are well established to positively influence CRF levels [98].

Exercise promotes cardiovascular health partly through its influence on endothelial function by increasing shear stress—a mechanical force exerted by flowing blood on the vascular wall [49, 99–103]. This shear stress is crucial for maintaining endothelial health, including attenuating oxidative stress and limiting endothelial senescence, a factor in aging and disease progression [104–106]. Healthy endothelia are less likely to adopt a senescent phenotype, which is characterized by changes that can directly impact cancer progression, such as alterations in the secretory functions of endothelial cells [107–111]. These cells play a vital role in modulating the tumor microenvironment by secreting growth factors and cytokines that can either suppress or support tumor growth [112–115]. Furthermore, robust endothelial function enhances barrier integrity, which may reduce the likelihood of cancer metastasis by limiting the extravasation of tumor cells [116–118]. However, in cases where endothelial health is compromised, a pro-inflammatory phenotype may prevail, potentially facilitating tumor progression and metastasis by disrupting normal cellular barriers and promoting an environment conducive to cancer cell migration and invasion [118–123]. Thus, through regular exercise-induced improvement of endothelial function, there is also a potential for modulation of cancer-related processes, highlighting a significant yet often overlooked pathway through which PA may influence cancer development and progression.

Regular PA and exercise can lead to long-term reductions in chronic inflammation, mediated through decreased production of pro-inflammatory cytokines and increased release of anti-inflammatory cytokines. Since chronic inflammation is a known risk factor for the development and progression of cancer, its reduction can potentially lower cancer risk [124, 125].

Engaging in regular PA has been closely associated with improved DNA repair capabilities and greater genomic stability [126–130]. This beneficial effect is critical, as it enhances the natural stress resilience mechanisms of the cells to repair DNA damage, which can occur due to environmental factors, lifestyle choices, and dietary factors. Enhanced DNA repair helps maintain the integrity of the genome, preventing the accumulation of harmful genetic mutations that are a primary driver of cancer development.

Regular PA stimulates key metabolic pathways that significantly enhance lipid oxidation and overall metabolic efficiency [56, 57, 131–136]. This metabolic enhancement extends to mitochondrial function, where exercise induces improvements in the efficiency and activity of mitochondria [136]. Improved mitochondrial function leads to a reduction in oxidative stress, thereby decreasing the likelihood of DNA mutations that can lead to cancer.

Regular PA and exercise are known to strengthen immune surveillance, a vital function that enhances the body’s ability to detect and eliminate cancerous cells at early stages [137]. This increased surveillance involves the activation and proliferation of various immune cells, such as natural killer (NK) cells and T-cells, which are crucial for identifying and destroying malignant cells before they can become established tumors. Furthermore, exercise not only boosts the quantity of these immune cells but also improves their functionality, thereby enhancing their ability to combat cancer effectively [138–141]. PA also triggers the mobilization of immune cells by stimulating the lymphatic system, which facilitates the circulation of immune cells throughout the body. This enhanced circulation may allow immune cells to reach and infiltrate tumors more effectively. The release of specific cytokines and growth factors during and after exercise also plays a role in modulating the immune response, further supporting the anti-cancer activity of the immune system.

Additionally, regular PA is a potent stress reducer known to decrease psychological stress levels through several biological pathways [142]. One primary mechanism is the release of endorphins. Additionally, PA helps modulate other neurotransmitters in the brain, such as serotonin and dopamine, which play significant roles in regulating mood and stress responses. The reduction of stress through PA can have significant implications for cancer risk and progression. Chronic stress is associated with elevated levels of cortisol, a stress hormone that, when consistently high, can weaken the immune system [143–147]. A compromised immune system is less capable of fighting off the initiation and progression of cancer cells. By reducing the levels of cortisol and other stress-related hormones [148, 149], exercise helps maintain a robust immune response, reducing the likelihood of cancerous growth and spread. Furthermore, lower stress levels are linked to better lifestyle choices, such as improved diet and sleep, which further decrease cancer risk. Through these mechanisms, regular PA serves as a crucial component in a comprehensive cancer prevention strategy, addressing both the physical and psychological dimensions of health.

Regular PA and exercise also significantly impact the circulating levels of other hormones that are closely associated with cancer risk [150]. For example, exercise has been shown to modulate circulating estrogen levels, a factor linked to a decreased risk of hormone-sensitive cancers such as breast and endometrial cancer. Additionally, regular PA can also lead to decreases in abnormally high levels of testosterone, which, when elevated, may increase the risk of certain cancers [151].

Regular PA and exercise are also associated with improved insulin sensitivity [152], which plays a crucial role in reducing cancer risk by influencing cell growth and proliferation. Enhanced insulin sensitivity helps modulate the insulin and insulin-like growth factor-1 (IGF-1) pathways, which are implicated in the progression of several cancers. Additionally, regular PA contributes to decreased obesity and central adiposity—factors strongly linked to an increased risk of site-specific cancers such as colorectal, breast, and endometrial cancers [150]. This reduction in body fat is particularly important as it also leads to a decrease in pro-inflammatory leptin and other obesity-related cytokines, which are known to promote a pro-carcinogenic environment. Simultaneously, PA induces a significant increase in anti-inflammatory adiponectin levels, further contributing to a systemic anti-inflammatory state [3].

PA and exercise are increasingly recognized for their role in epigenetic regulation [153–157], which involves changes in gene expression that do not alter the DNA sequence but can significantly influence cellular function and health. PA affects the epigenome through various mechanisms, including DNA methylation, histone modification, and the regulation of non-coding RNA [155–157]. These epigenetic alterations can activate or suppress gene expression, leading to changes in the pathways involved in cell growth, apoptosis, and DNA repair—all of which are crucial for cancer prevention and control. For example, exercise-induced alterations in DNA methylation at certain gene sites can lead to the reactivation of tumor suppressor genes and the inhibition of oncogene expression [158]. Similarly, modifications in histone acetylation and methylation can enhance the accessibility of transcription factors to DNA, promoting the expression of genes that protect against cancer. Furthermore, exercise influences the expression of microRNAs, which are involved in post-transcriptional regulation of gene expression [159, 160], potentially leading to decreased inflammation and reduced tumor growth. Through these epigenetic modifications, regular PA and exercise create a cellular environment that can thwart the initiation and progression of cancer, illustrating a powerful, yet underappreciated, pathway through which lifestyle factors that alter CRF can influence cancer risk.

Improved CRF via regular PA may reduce the risk of lung cancer via the increased functional capacity of the lung, improved antioxidant defense, decreased concentrations of carcinogenic metabolites (e.g., produced from smoking), and increased ventilation and perfusion, which may reduce the interaction time and concentrations of any carcinogenic agents in the airways [161–163].

For colorectal cancer, it has been postulated PA increases gut motility and levels of prostaglandins, which reduce the gastro-intestinal transit time; this process subsequently reduces the contact time between fecal carcinogenesis and the colonic mucosa and allows less opportunity for carcinogenesis [164].

The relationship, however, between CRF and cancer is not uniformly protective. The evidence for prostate cancer and hematological cancers like leukemia and myeloma were not conclusive but also suggest a potentially increased risk of these cancers with high CRF levels (Fig. 1). The inconclusive findings for prostate cancer reflect those of previous studies that have reported on the associations of PA with prostate cancer [165, 166]. These potential positive associations may involve complex interactions between exercise-induced hormonal changes, such as increased levels of testosterone, which could potentially stimulate prostate cancer growth [167, 168]. Exercise stimulates the production of IGF-1 [169], which modulates cell growth and survival, and has been shown to increase the risk of prostate cancer [170, 171]. Exercise is known to increase blood flow and promote angiogenesis [172], which is generally beneficial for tissue health and healing, but might facilitate the growth of tumor cells through an increased supply of nutrients and oxygen [173]. Another biological plausibility for the positive association between CRF and prostate cancer could also be via dehydroepiandrosterone sulfate, an adrenal androgen which is related to physical fitness [174], and has been shown to promote prostate cancer [175]. In addition to these pathways in influencing the potentially increased risk of prostate cancer observed with high CRF levels, increased healthcare awareness and screening and early detection have also been implicated [68, 176]. However, findings of the FIT project suggested that although men with high CRF levels are more likely to undergo PSA screening, this does not account for the increased incidence of prostate cancer observed in these individuals [83]. Studies that reported positive associations between CRF and prostate cancer risk have generally been characterized by short follow-up durations (< 10 years), whereas studies that have demonstrated no associations had long follow-up durations [35]. Hence, reverse causation bias may be another potential explanation for these findings, given that many cancers including prostate cancer have a long subclinical development which may cause PA and CRF to decline in the early stages of follow-up [35]. No associations were observed for CNS cancer, thyroid cancer, esophageal cancer, and Hodgkin’s lymphoma, and the evidence for CRF’s impact on the risk of skin, mouth and pharynx, kidney, and endometrial cancers is limited and inconclusive (Fig. 1), suggesting that the relationship between CRF and cancer risk might be cancer-specific and influenced by a variety of genetic, environmental, and lifestyle factors.

The observations from studies that report no significant association or even an increased risk of leukemia and myeloma with higher levels of CRF are intriguing, especially in light of the generally protective effects of higher CRF against most other types of cancer. Several factors including theoretical mechanisms could potentially explain these paradoxical findings. Intense and prolonged PA can lead to increased production of reactive oxygen species (ROS) and oxidative stress [177]. While moderate exercise typically enhances antioxidant defenses, excessive ROS generation from high-intensity exercise can potentially cause DNA damage and contribute to carcinogenesis, including leukemia [178]. Excessive or high-intensity exercise can induce chronic inflammatory responses [179], which have been linked to the development of various cancers, including hematologic malignancies like leukemia [180]. Intense PA may transiently suppress the immune system [181], potentially reducing its ability to detect and eliminate malignant cells. This immunosuppressive effect could theoretically allow for the proliferation of pre-leukemic cells. PA impacts bone turnover and the bone marrow microenvironment [182]. Alterations in this microenvironment due to intense exercise might influence hematopoietic stem cells and potentially lead to leukemogenesis. Myeloma, unlike solid tumors, originates in the bone marrow from plasma cells [183]. The unique microenvironment of the bone marrow [184–187], which includes interactions between plasma cells and the bone marrow stroma, cytokines, and growth factors, may respond differently to the physiological effects of PA, exercise, and high CRF [188–190]. The bone marrow microenvironment plays a crucial role in the progression of multiple myeloma by secreting a range of cytokines and growth factors (e.g., IL-6, VEGF, TGF-β, IGF-1, CXCL12) that support the survival, proliferation, and resistance to apoptosis of myeloma cells [191]. Interestingly, many of these cytokines and chemokines are also released from skeletal muscle during exercise, known as “exerkines,” which can influence systemic inflammation and immune regulation [59–61]. Regular PA and/or exercise influences the immune system significantly, typically enhancing surveillance and reducing cancer risk [192]. However, in the case of myeloma, exercise-induced immunological changes might inadvertently support the growth or survival of malignant plasma cells [181]. For instance, certain cytokines or growth factors that are beneficial in controlling other cancers might promote the survival or proliferation of myeloma cells due to their specific biological characteristics. Furthermore, high levels of PA and CRF are associated with changes in hormone levels and metabolic states that generally protect against cancer. However, specific changes in the hormonal or metabolic milieu due to high CRF could potentially influence these hematological cancers differently than other cancers and thereby could favor the development or progression of these cancers. Individual factors such as the type, intensity, and duration of exercise, as well as the individual’s overall health status, genetic predispositions, and existing medical conditions, may also play a role. Overall, the research evidence indicates that the benefits of regular, moderate exercise far outweigh the potential risks related to cancer growth. In conclusion, CRF appears to play a significant role in reducing the risk of several cancers through various biological mechanisms, including inflammation reduction, immune system enhancement, hormonal regulation, and metabolic improvements.

## Clinical and public health implications

The comprehensive findings showing that high CRF is associated with a lower risk of various cancers, including head and neck, lung, breast, gastrointestinal particularly pancreatic and colorectal, bladder, overall cancer incidence, and mortality, alongside a potential decreased risk of stomach and liver, bile duct, gall bladder cancers, underscores the significance of CRF as a pivotal factor in cancer prevention and management. More profoundly, CRF not only reflects an individual’s capacity to perform physical activities but also encapsulates broader effects on the basic biology of aging. This connection highlights that improvements in CRF are linked to fundamental biological mechanisms that deter age-related declines and maladaptations, which are often precursors to cancer. Therefore, enhancing CRF through regular PA offers a vital, accessible strategy for extending health span and reducing cancer risk, reinforcing the need for public health initiatives that promote physical fitness across all ages.

These findings may have several critical clinical and public health implications. The protective effect of high CRF against multiple cancer types emphasizes the need for healthcare providers to advocate for and integrate PA and/or exercise training into preventive and therapeutic strategies for patients across all demographics [193]. We observed that generally CRF levels > 7 METs may offer protection against specific cancers; these findings could serve as a basis for developing clinical guidelines that recommend target CRF levels for cancer prevention. It is well known CRF levels generally decline in later life due to factors such as aging, comorbidities, and decreased participation in PA [48]. Furthermore, adjuvant treatments for cancer, such as chemotherapy and radiation, can cause declines in CRF levels [194]. These underscore the importance of identifying or developing interventions aimed at maintaining or improving CRF in older individuals as well as patients with cancer. Indeed, a few reports suggested that improving levels of CRF over time could significantly reduce the likelihood of developing some cancers as well as cancer mortality [84, 87]. Given the consistent inverse relationship between CRF and cancer outcomes across age, sex, and race, CRF assessment could be utilized in personalized medicine approaches to identify individuals at higher risk and tailor prevention and treatment strategies accordingly. The inconclusive evidence regarding CRF’s association with skin, mouth and pharynx, kidney, and endometrial cancers highlights areas for further research to understand these relationships better and possibly expand the range of cancers influenced by CRF. Overall, these findings suggest that enhancing CRF through regular PA and/or exercise training could be a key strategy in cancer prevention and survivorship care, advocating for broader public health initiatives aimed at increasing overall fitness across populations.

## Interventions to improve CRF levels

To address the decline in CRF levels associated with aging, comorbidities, and the adverse effects of cancer treatments such as chemotherapy, hormone therapy, and radiation, there is a need to implement, identify, or develop interventions aimed at maintaining or improving CRF in these populations. The importance of maintaining or enhancing CRF extends beyond general health, playing a pivotal role in cancer prevention, potential recurrence mitigation, and enhancing the quality of life for those diagnosed with cancer. Although CRF is determined by many factors that cannot be modified such as age, sex, and genetics, it remains a modifiable risk factor [42]. CRF has a strong genetic component with an estimated heritability of 40–70% [195–197]. However, it is well established that regular PA and/or exercise training is an effective intervention to improve CRF levels. An absolute CRF level of ≤ 5 METs has been consistently shown to be associated with the worst prognosis [198, 199]. Although the precise amount of PA necessary to attain specific CRF levels remains uncertain, it is known that adherence to moderate-intensity exercise training guidelines typically enables middle-aged individuals to reach or exceed moderate CRF levels (> 8 METs) [200, 201]. Based on a large-scale prospective cohort study in which habits of PA were assessed using questionnaires, optimal CRF levels (age-standardized METs: 9 in men, 7 in women) were reported to approximate to about 130 min/week and 148 min/week of brisk walking (8.2 MET-hr/wk and 9.4 MET-hr/wk) for men and women, respectively [202]. It has also been reported that an exercise capacity > 5 METs can be achieved by regularly exercising > 3 METs (which corresponds to moderate-to-vigorous PA) [203]. Guidelines such as the American Cancer Society (ACS) Guideline for Diet and Physical Activity for Cancer Prevention recommend 150–300 min of moderate-intensity or 75–150 min of vigorous-intensity activity each week (or a combination of these) for cancer prevention [204]. High-intensity exercise training has emerged as a potent strategy to increase levels of VO2peak [205, 206], especially in cancer patients whose levels often fall below the requisite threshold [207]. Based on a consensus from various health organizations, guideline bodies, and research findings, exercise recommendations for cancer survivors are as follows: Engage in moderate-intensity aerobic training, such as walking, 3–4 times/week for 30–40 min/session. It is suggested to aim for a cumulative total of 150–300 min of moderate activity each week, or 75–150 min if engaging in vigorous-intensity activities. This can be broken down into several sessions lasting ≥ 10 min each. Furthermore, incorporate full body strength training into your routine 2 days/week. A strength training program can include exercises that work for major muscle groups, supporting muscle strength and bone health, which are especially important for cancer survivors [208]. While PA and exercise are widely recognized as major contributors to improving CRF levels, research has shown that the response to these interventions can vary significantly among individuals and/or may not be universally effective for everyone [209]. This variability is often due to genetic factors [210], which can account for more than 50% of the individual differences in CRF [195–197]. Moreover, the genetic predisposition that affects CRF also suggests that improvements in CRF might not directly translate to reduced cancer risk for everyone. It is tempting to say that given CRF’s large genetic component, and the large amount of evidence being observational, it may be that the inverse relationships between CRF and some cancer types are due to inherent genetic differences and not necessarily CRF and therefore improving CRF may not actually reduce risk. However, given that the observational evidence linking increased CRF levels to reduced risk of certain cancers aligns strongly with the Bradford Hill criteria for causality [211], this reinforces the notion that higher CRF may indeed confer a protective effect against certain cancer types across the general population. Despite the potential influence of genetic differences, the consistency and strength of these associations underscore a likely beneficial impact of improving CRF. However, MR studies, which could provide more definitive evidence of causality, remain limited in this area. This scarcity is primarily due to the challenges in identifying specific genetic variants that accurately reflect CRF levels, complicating efforts to fully disentangle the genetic contributions from the observed health outcomes.

There are potential alternative methods that could be used to enhance CRF levels. Complementing exercise with nutrition therapy may further augment CRF improvements. Lifestyle interventions that combine dietary counseling with tailored exercise training have demonstrated significant improvements in VO2peak among cancer survivors [212, 213], suggesting a synergistic effect that transcends the benefits of exercise alone. Dietary supplementation with amino acids, n-3 polyunsaturated fatty acids, and L-carnosine, hypocaloric diet, and dietary patterns such as Dietary Approaches to Stop Hypertension (DASH) and Mediterranean diets may improve levels of CRF, but the evidence is limited [214]. Nutrition therapy has a potential role to play in increasing CRF in populations with exercise limitations such as cancer survivors, but more research is needed. In certain scenarios, pharmacological interventions may complement lifestyle modifications to improve CRF levels. Although not as extensively studied as exercise, emerging research suggests that specific medications might facilitate CRF improvements, warranting further investigation. The use of medications such as renin–angiotensin–aldosterone system inhibitors, hydralazine, and digoxin in populations with impaired CRF, such as heart failure, have been shown to increase levels of VO2peak [215, 216]. While exercise, diet, and potential medications are essential components of enhancing CRF, addressing other modifiable factors such as smoking, body composition, and weight, which strongly influence CRF levels, may provide a more comprehensive approach to improving CRF. Smoking has a well-documented negative impact on lung function, overall cardiovascular health, and subsequently fitness levels [217, 218]. Programs that include smoking cessation have been shown to significantly reduce tobacco-induced cardiovascular damage and improve overall CRF levels within a few months [219]. Encouraging individuals to quit smoking can lead to substantial improvements in CRF, lung capacity, and cardiovascular response to exercise. Altering body composition, primarily through reducing fat mass and increasing muscle mass, directly influences CRF [220]. Strategies such as resistance training to increase lean mass and cardiovascular training to decrease fat mass can improve overall body composition, thereby enhancing oxygen utilization efficiency and CRF [221]. Excess body weight, particularly obesity, places additional strain on the cardiovascular system; achieving and maintaining a healthy weight is, therefore, essential for improving CRF. Weight loss, as facilitated by combining dietary modifications with consistent PA, can significantly enhance CRF by improving heart function and reducing the metabolic load on the cardiovascular system [222]. In summary, interventions aimed at boosting CRF should ideally integrate exercise training with nutrition therapy and, where applicable, other non-exercise-related strategies discussed above. On the other hand, these strategies could serve as alternative options for those who have limited response to conventional exercise. Tailoring these interventions to individual needs and limitations is paramount, especially for older adults and individuals with cancer, to counteract the age-related and treatment-induced declines in CRF. Furthermore, interventions need to be tailored to the individual’s genetic background and lifestyle factors.

## Gaps and future directions

The impact of CRF on cancer outcomes presents a fertile ground for future research, driven by the preliminary findings and gaps identified in existing studies. Several critical research areas are essential to advancing our understanding and application of CRF in cancer prevention and management. Most studies considered only baseline assessments of CRF. A few reports suggested that improving levels of CRF over time could significantly reduce the likelihood of developing some cancers [84, 87]. Hence, future studies should incorporate repeated CRF assessments over time to mitigate regression dilution bias and more accurately capture the dynamic nature of CRF and its impact on cancer risk. We have shown in our reproducibility studies of CRF within the KIHD cohort that CRF exhibits substantial within-person variability (regression dilution ratio = 0.58) [7, 10, 35, 223]; hence, use of baseline measurements can underestimate the extent of the true association between CRF and outcomes. Accounting for changes in CRF eliminates or minimizes the influence of genetics on CRF, given that CRF changes are primarily the result of the individual’s PA and sedentary behaviors [193].

The inconclusive evidence regarding CRF’s relationship with several site-specific cancers such as skin, mouth and pharynx, kidney, and endometrial cancers necessitates further large-scale longitudinal studies. These investigations could provide a clearer picture of CRF’s potential protective effects across a broader spectrum of cancers. There is a need for in-depth research to explore the complex associations between high CRF and potentially increased risks of prostate and hematological cancers. Studies should consider and account for factors like healthcare awareness, screening practices, and reverse causation bias. Findings for some site-specific cancers (e.g., hematological cancers, endometrial cancer, mouth and pharynx cancer, thyroid cancer) were based on only one or two cohorts; hence, more research is needed to evaluate these cancers. Identifying the precise CRF levels that confer protection against various cancers is crucial. Detailed dose–response studies could help establish targeted CRF benchmarks for cancer prevention. There are no direct studies focusing specifically on CRF and cancer recurrence; research in this area may provide crucial insights into how interventions aimed at improving CRF can be utilized to potentially lower the risk of cancer recurrence. The paucity of MR studies on CRF and cancer outcomes highlights the necessity for large-scale GWAS. These studies aim to identify genetic variants that accurately reflect CRF levels, paving the way for MR studies to help untangle the causal pathways between CRF and specific types of cancer. Understanding the biological mechanisms through which CRF exerts its protective effects against cancer is fundamental. Mechanistic studies can uncover the pathways involved, potentially identifying CRF as a therapeutic target for cancer prevention. By addressing these future directions, the scientific community can build upon the existing evidence, enhancing our understanding of CRF’s role in cancer prevention and therapy and ultimately guiding health recommendations and interventions aimed at cancer prevention through improved CRF.

## Conclusions

The current body of evidence on CRF and its relationships with various cancer outcomes present a complex but generally positive picture. The findings underscore the pivotal role of CRF in cancer prevention and survivorship. High CRF levels were linked to a notably lower risk of several major cancers, including head and neck, lung, breast, gastrointestinal particularly pancreatic and colorectal, bladder, overall cancer incidence and mortality, and potentially stomach and liver, bile duct, and gall bladder cancers. This protective effect, consistent across different demographics, suggests that CRF may serve as a universal marker of reduced cancer risk and improved outcomes following a cancer diagnosis. The identification of a potential CRF threshold (> 7 METs) that confers protection for some cancer endpoints, a threshold approximately consistent with that for adverse cardiovascular outcomes (> 8 METs) [224], adds a quantifiable target for public health initiatives and individual fitness goals. However, the relationship between CRF and cancer is not uniformly protective, with high CRF levels putatively associated with a higher risk of prostate cancer and certain hematological cancers, a research area which needs urgent exploration. Future research should focus on large-scale longitudinal and genetic studies to further elucidate the causal relationships and mechanistic pathways linking CRF to cancer outcomes. Addressing the gaps in evidence for certain cancers and exploring the detailed relationships between CRF and cancer risk will refine our understanding and guidance on using CRF as a preventive and therapeutic tool against cancer.

## Supplementary Information

Below is the link to the electronic supplementary material.

Supplementary file1 (DOCX 25 KB)

## Data Availability

This is a narrative review; no new scientific data was generated, and all data are within the paper.
