# Supplementary material for: Unraveling the link between cardiorespiratory fitness and cancer: a state-of-the-art review
Source: GeroScience. 2024 Jun 3;46(6):5559–85. doi: 10.1007/s11357-024-01222-z (PMC11493895; doi:10.1007/s11357-024-01222-z)
Supplement: Supplementary file 1 — Supplementary file1 (DOCX 25 KB) [file 11357_2024_1222_MOESM1_ESM.docx]

**Supplementary Material.** Key studies and meta-analyses that have evaluated the associations of cardiorespiratory fitness with prostate cancer

| **Author, year of publication [reference]** | **Country** | **Study design** | **Assessment method for CRF** | **Number of studies / Events / Participants** | **Follow-up, yrs** | **Results (Multivariable adjusted estimates)** | **Summary of findings** |
| --- | --- | --- | --- | --- | --- | --- | --- |
| Oliveria, 1996 | USA | Prospective cohort | Maximal exercise treadmill test | 1 / 94 / 12975 | NR | (HR=0.26, 95% CI, 1.10-0.63) comparing the top vs bottom quartiles of CRF | Inverse association between CRF and prostate cancer |
| Byun, 2011 | USA | Prospective cohort | Symptom-limited exercise treadmill testing | 1 / 634 / 19042 | 9.3 | (HR=1.68, 95% CI, 1.13-2.48) and (HR=1.74, 95% CI, 1.15-2.62) for moderate and high CRF categories, respectively, compared to the lowest CRF category. | **High CRF associated with increased risk of prostate cancer** |
| Lakoski, 2015 | USA | Prospective cohort | Indirect from incremental treadmill test | 1 / 1310 / 13949 | 6.5 | (HR=1.22, 95% CI, 1.02-1.46) comparing high vs low CRF categories. | **High CRF associated with increased risk of prostate cancer** |
| Robsahm, 2017 | Norway | Prospective cohort | Indirect from incremental cycle test | 1 / 213 / 1997 | 26.2 | (HR=1.20, 95% CI, 0.83-1.74) comparing the top vs bottom tertile | No evidence of an association |
| Steell, 2019 | UK | Prospective cohort | Submaximal cycle ergometer test | 1 / 436 / 73259 | 5.0 | (HR=1.16, 95% CI, 1.02-1.32) comparing high CRF (>10 METs) with average CRF | **High CRF associated with increased risk of prostate cancer** |
| Crump, 2020 | Sweden | Prospective cohort | Indirect from workload on ergometer | Prostate cancer: 1 / 10782 / 699125  Aggressive events: 1817  Mortality events: 217 | 38.8 million person-years | (HR=1.10, 95% CI, 1.03-1.19) for any prostate cancer, (HR=1.01, 95% CI, 0.85-1.21) for aggressive prostate cancer, and (HR=1.24, 95% CI, 0.73-2.13) for prostate cancer mortality, comparing high vs low CRF levels | **High CRF associated with increased risk of prostate cancer.** |
| Reiter-Brennan, 2021 | USA | Retrospective cohort | Indirect from workload on treadmill | 1 / 739 / 22827 | 7.5 | (HR=1.80, 95% CI, 1.27-2.54) for prostate cancer incidence in men >55 years and (HR=0.40, 95% CI, 0.19-0.86) for prostate cancer mortality comparing high CRF (≥12 METs) vs low CRF (<6 METs) | **High CRF associated with increased risk of prostate cancer incidence in men >55 years** and decreased risk of prostate cancer mortality |
| Kunutsor, 2021 | Finland | Prospective cohort and systematic review | Respiratory gas exchange analysis | Cohort: 1 / 216 / 2204  Review: 8 studies | Cohort: 24.9  Review: 5.0-44.0 | Cohort: (HR=1.28, 95% CI, 0.87-1.88) comparing the top vs bottom tertiles of CRF  Review: Mixed evidence. Studies reporting positive associations had short-term follow-up durations (<10 years). | No evidence of an association |
| Ekblom-Bak, 2023 | Sweden | Prospective cohort | Submaximal cycle ergometer test | 1 / 1918 / 177709 | 9.6 | (HR=1.18, 95% CI, 1.02-1.38) for prostate cancer incidence comparing moderate CRF (10-13 METs) with very low CRF (≤10 METs) levels  Risk of prostate cancer mortality decreased continuously across CRF range 7-13 METs | **High CRF associated with increased risk of prostate cancer incidence** and decreased risk of prostate cancer mortality |
| Vainshelboim, 2023 | USA | Prospective cohort | Non-exercise based equation | 1 / 26096 / 402548 | 13.7 | (HR=1.09, 95% CI, 1.00-1.20) comparing higher (>10.9 METs) vs lower categories (<8.9 METs) of CRF | **High CRF associated with increased risk of prostate cancer** |
| Wang, 2023 | Norway | Prospective cohort | Non-exercise based equation | 1 / 1376 / 46968 | 22.1 | (HR=0.85, 95% CI, 0.72-1.02) comparing the highest vs lowest CRF category | Inverse association between CRF and prostate cancer |
| Onerup, 2023 | Sweden | Prospective cohort | Indirect from workload on cycle ergometer | Incidence: 1 / 14232 / 1078000  Mortality: 1 / 948 / 19686 | Incidence: 33  Mortality: 6.5 | Prostate cancer incidence: (HR=1.07, 95% CI, 1.03-1.12) comparing high vs low CRF categories.  Prostate cancer mortality:  (HR=0.84, 95% CI, 0.70-1.02) comparing high vs low CRF categories. | **High CRF associated with increased risk of prostate cancer incidence** and no association with prostate cancer mortality |
| Bolam, 2024 | Sweden | Prospective cohort | Submaximal cycle ergometer test | Incidence: 1 / 592 / 57652  Mortality: 1 / 46 / 592 | 6.7 | Prostate cancer incidence: (HR=0.65, 95% CI, 0.49-0.86) per increase in annual CRF by +3% compared with decreased CRF -3%  Prostate cancer mortality:  (HR=0.98, 95% CI, 0.94-1.02) per increase in CRF | Inverse association between change in CRF and prostate cancer incidence, and no association for prostate cancer mortality |

CI, confidence interval; CRF, cardiorespiratory fitness; HR, hazard ratio; MET, metabolic equivalent; NR, not reported
